# New microsatellite loci for the green sea urchin *Strongylocentrotus droebachiensis* using universal M13 labelled markers

**DOI:** 10.1186/1756-0500-7-699

**Published:** 2014-10-07

**Authors:** Marc B Anglès d’Auriac, Anders Hobæk, Hartvig Christie, Hege Gundersen, Camilla With Fagerli, Johannes Haugstetter, Kjell Magnus Norderhaug

**Affiliations:** Norwegian Institute for Water Research, N-0349 Oslo, Norway; Norwegian Institute for Water Research, N-5006 Bergen, Norway; ecogenics GmbH, 8952 Schlieren, Switzerland; Department of Biosciences, University of Oslo, Box 1066, Blindern, 0316 OSLO, Norway

**Keywords:** Climate change, Heterozygote deficiency, Kelp forests, Marine invertebrates, Microsatellites, NE Atlantic, Simple DNA preparation, *Strongylocentrotus droebachiensis*

## Abstract

**Background:**

The green sea urchin *Strongylocentrotus droebachiensis* has a wide circumpolar distribution and plays a key role in coastal ecosystems worldwide by destructively grazing macroalgae beds and turn them into marine deserts, so-called barren grounds. In the past decades, large established kelp forests have been overgrazed and transformed to such barren grounds on the Norwegian coast. This has important repercussions for the coastal diversity and production, including reproduction of several fish species relying on the kelp forests as nurseries. Genetic diversity is an important parameter for the study and further anticipation of this large scale phenomenon.

**Findings:**

Microsatellites were developed using a Norwegian *S. droebachiensis* individual primarily for the study of Northeast Atlantic populations. The 10 new microsatellite loci were amplified using M13 forward tails, enabling the use of M13 fluorescent tagged primers for multiplex reading. Among these loci, 2 acted polysomic and should therefore not be considered useful for population genetic analysis. We screened 96 individuals sampled from 4 different sites along the Norwegian coast which have shown unexpected diversity.

**Conclusions:**

The new microsatellite loci should be a useful resource for further research into connectivity among *S. droebachiensis* populations, and assessing the risks for spreading and new overgrazing events.

## Findings

The green sea urchin *Strongylocentrotus droebachiensis* is one of the dominant grazing species in temperate marine ecosystems. Catastrophic overgrazing events have been recorded in the Pacific [1] and the Atlantic [2] including the northern Norwegian and Russian coast, where approximately 2000 km^2^ of kelp forest were grazed to barren grounds in the 1970s [3, 4]. Because of the persistence of the sea urchin dominance and the large loss of biodiversity and production, it is important to develop new genetic tools for studying and improving our understanding of this species. Microsatellite loci have been previously developed using North American individuals [5]. Further analysis have shown that the Northeast Atlantic population, based on one sampling location in Iceland and one in Norway, is differentiated compared from Northwest Atlantic and Pacific populations [6]. Moreover, when tested on the two Northeast Atlantic locations, locus Sd76 was reported failing to amplify any of the individuals and the remaining 3 loci showed fewer alleles compared with North American locations. These results have motivated our goal to develop the present microsatellite loci using a *S. droebachiensis* individual from a Norwegian population (Drøbak) in an effort to increase genetic information produced for the study, primarily, of the Northeast Atlantic populations.

Sea urchins were sampled by SCUBA from four stations along a wide geographical area along the Norwegian coast from Drøbak in the south (59.7°N) to Veidnes in Finnmark in the north (70.8°N) (Table 1). Sea urchins were sampled within frames haphazardly placed on the sea floor. All urchins within each frame were sampled until (at least) 30 sea urchins were collected. Gonad tissue from each individual was stored in vials with 96% ethanol. Sampling was performed according to Norwegian laws. The field sampling and handling of *S. droebachiensis*, a non-threatened species, did not conflict with any national or international legislation.

**Table 1:** **Sample information**

County	Area	Station	Code	Depth	Lat. (°N)	Long. (°E)
Akershus	Oslofjord	Drøbak	D2	20	59.66	10.63
Nordland	Torghatten	Helløya	NH	5	65.38	12.01
Nordland	Vega, nord	Skogsholmen	NS	5	65.81	12.04
Finnmark	Kongsfjord	Veidnes	FV	5	70.70	29.44

Microsatellite sequences were isolated by ecogenics GmbH (Zurich, Switzerland), based on an individual collected at the type locality of this species (Drøbak, Oslofjord) more than 200 years after it was described there for the first time [7]. Size-selected fragments from genomic DNA were enriched for Simple Sequence Repeat (SSR) content by using magnetic streptavidin beads and biotin-labelled CT and GT repeat oligonucleotides. The SSR-enriched library was analysed on a Roche 454 platform using the GS FLX Titanium reagents. The total 7,840 reads had an average length of 202 base pairs. Of these, 355 contained a microsatellite insert with a tetra- or a trinucleotide of at least 6 repeat units or a dinucleotide of at least 10 repeat units. Suitable primer design was possible in 125 reads, of which 28 were tested for functionality and polymorphism. Among these 28 candidate loci and associated primers for PCR, 8 did not amplify, one was monomorphic, four were too weak, three were too difficult to interpret, two showed more than two alleles in some samples, resulting in the selection of 10 loci and primer pairs (Table 2). Polymorphism was tested in 15 individuals from various locations.

**Table 2:** **Characterization of 10 microsatellite loci for** ***S. droebachiensis*** **using 96 individuals**

Locus	Repeat motif	F & R primer sequences 5’-3’*	Dye	Size range (bp)**	***A***	***N***	***H***_o_	***H***_e_
Strdro-97	(CA)_11_	F: GGTGCATCCATCCCGTAGTG	FAM	159-171	6	96	0.29	0.34
		R: CTGCCCTGTAAGAGTGTGTG						
Strdro-837***	(CA)_11_	F: GAGAGTGAATTGAATGTATCAAATGAG	Yakima Yellow	142-152	6	67		
		R: TTTATCCCACAGGACTCGGC						
Strdro-849***	(AC)_12_	F: TGATACACGTCTGTGAAAACCC	ATTO 550	112-154	15	94		
		R: TTTGCTCCATCAGAAGTTCAC						
Strdro-1051	(GA)_11_	F: GCGCTAGTCTGTTTCACCAC	ATTO 565	190-218	13	95	0.37	0.68
		R: TCAAATTCCGCCGTTTAGGC						
Strdro-4147	(GA)_11_	F: GTCAGACAAAGAGAGTGTGTGAG	FAM	75-115	15	84	0.30	0.82
		R: GAGTATATGCCCGCCTCTCC						
Strdro-7209	(CA)_12_	F: TGAACTGGCCACATTCCTCC	Yakima Yellow	128-158	9	82	0.40	0.41
		R: ACTATGTGGGGGTAGATCCG						
Strdro-5563	(ATGG)_11_	F: TGTTCAGCTGCCTGTCTCTG	ATTO 550	165-247	19	90	0.48	0.85
		R: TCCTTTTGTTCATCCTTCCTTCC						
Strdro-1356	(CA)_12_	F: AAAAGGTAACGTTCGCTCGC	ATTO 565	164-250	19	87	0.33	0.77
		R: ATGATCCGTTCAGGAGGCAG						
Strdro-7412	(GA)_13_	F: ACGATTGGGAGATTGAAAGTG	FAM	92-124	13	95	0.68	0.80
		R: TCGCCACATACACACAAACG						
Strdro-5950	(GT)_11_… (GT)_12_	F: GATTGAACCGTGTGCGAGAG	ATTO 565	96-152	19	89	0.44	0.89
		R: CTACCTCCAACGACACACAC						

Number of alleles (*A*), number of individuals that amplified (*N*), observed heterozygosity (*Ho*), expected heterozygosity (*He*), *without M13 forward tail, **including the 18 bp M13 forward tail, ***acts as polysomic.

A simple optimised DNA extraction was performed using QuickExtract (Epicentre Technologies Corporation, Madison, USA). Briefly, 10 to 20 mg gonad material from one individual preserved in Ethanol 96% was washed in distilled water prior to adding 100 μL QuickExtract buffer. Samples were incubated at 65°C for 10 min followed by 98°C inactivation for 5 min. The lysates were further diluted 10^-2^ in Tris EDTA buffer (Fluka Chemie GmbH, Buchs, Switzerland) prior to performing PCR. A 3-primer PCR approach using a M13 tail (5’-TGTAAAACGACGGCCAGT) for the forward primer was used for microsatellite loci amplification at concentrations as described previously [8]. Four different dyes were used for the universal M13 forward primer to enable fragment analysis multiplexing [9]. Simplex PCR amplifications, targeting one locus at a time, were performed using a CFX96 thermocycler (Bio-Rad, Hercules, CA, USA) in 10 μL reaction volume containing 5 μL iProof Master Mix (Bio-Rad), 0.04 μM of the forward primer with M13 5’-tail and 0.16 μM of each reverse and forward tagged M13 primers (Eurofins MWG, Ebersberg, Germany) and 2.5 μL sample. Reaction volume was completed with sterile deionised water. PCR amplifications were optimized and carried out under the following conditions: a denaturing step for 1 min at 98°C, followed by 30 cycles of 98°C for 5 s, 62°C for 10 s and 72°C for 15 s followed by 8 cycles of 98°C for 5 s, 57°C for 10 s and 72°C for 15 s. Up to 4 different simplex PCR plates, each with a different dye (Table 2), were mixed and diluted by transferring 5 μL each to a plate prefilled with 100 μL deionized water per well. From this dilution plate 1.2 μL per sample was transferred to the run plate prefilled with 10 μL Hi-Di Formamide (Applied Biosystems, Foster City, CA, USA) and 40% strength orange standard (MCLAB, San Francisco, CA, USA). PCR product sizes were determined using a 3730XL DNA analyser (Applied Biosystems) and scored using GeneMapper software version 4.0 (Applied Biosystems). GenAlEx software version 6.5 was used to report overall observed (*H*_o_) and expected (*H*_e_) heterozygosity [10]. Linkage disequilibrium and Hardy-Weinberg equilibria (HWE) were tested in Arlequin software version 3.5.1.3 [11].

We observed linkage (Likelihood ratio test, p < 0.05) among loci in all four populations, but no pair of loci was linked in all of them. Five loci (Strdro-1051, Strdro-1356, Strdro-4147, Strdro-5563, and Strdro-5590) showed heterozygote deficiencies and HWE deviations (Exact test, p < 0.05) in all populations, possibly suggesting inbreeding and substructuring or the presence of null-alleles. The fact that simplex PCR was performed for all loci strongly reduces the risk for weak amplification that may be observed in a multiplex assay and hence reduces possible false negative loci amplification results.

The loci presented in this study show higher allelic diversity than that reported previously for Northeast Atlantic populations using loci developed on Northwest Atlantic individuals [6]. Interestingly, heterozygote deficiencies and significant HWE deviation were also found with 3 of the 4 microsatellites used in this prior study [6], hence suggesting that *S. droebachiensis* may naturally deviate from HWE as it has been reported to be the case for many other marine invertebrates [12]. We believe that these new loci will be useful for the study of *S. droebachiensis*, in particular for the Northeast populations, for better monitoring the observed ongoing population distribution and densities fluctuations along the Norwegian coastline.

### Availability of supporting data

The microsatellite sequences are available through the European Molecular Biology Laboratory European Nucleotide Archive (http://www.ebi.ac.uk/ena/) Accession Numbers HG417080 to HG417089.
